# Recommendations for Anti-inflammatory Treatments in Alzheimer’s Disease: A Comprehensive Review of the Literature

**DOI:** 10.7759/cureus.4620

**Published:** 2019-05-08

**Authors:** Muhammad Mohsin Ali, Raza G Ghouri, Armghan H Ans, Arshia Akbar, Ahmed Toheed

**Affiliations:** 1 Internal Medicine, Mayo Hospital, King Edward Medical University, Lahore, PAK; 2 Cardiology, University of Pennsylvania, Philadelphia, USA; 3 Internal Medicine, Rawalpindi Medical College, Rawalpindi, PAK

**Keywords:** dementia, alzheimer disease, anti-inflammatory agents, non-steroidal anti-inflammatory drugs (nsaid), cognitive impairment, degenerative disease, neuroinflammation, disease prevention

## Abstract

Alzheimer’s disease (AD) is the most common cause of dementia in elderly patients, affecting individuals older than 60 years. It is a complex degenerative brain disease characterized by progressive cognitive impairment. AD constitutes a major global health concern. A central role for inflammation has been implicated in the pathogenesis of AD. Despite the understanding of multiple molecular pathways in the pathophysiology of AD, novel treatment agents with a possible role in modifying the disease activity are still lacking. Our article provides a comprehensive review of various observational studies and randomized trials encompassing the use of anti-inflammatory agents in the management of AD patients and utilizes the conclusions derived therefrom to give recommendations in this regard.

## Introduction and background

Alzheimer’s disease (AD) is the most common cause of dementing illnesses in elderly patients [1]. It is a degenerative brain disease, presenting with the characteristic features of cognitive difficulties, memory problems; challenges with learning and problem solving; and ultimately difficulty in speaking, swallowing, and walking [2]. A central role for inflammation has been implicated in the pathogenesis of AD [3]. This idea is evidenced by the increased activity of microglial cells, correlative with the severity of symptoms; as well as role of various inflammatory mediators in neurodegeneration, dysfunction of glial supportive cells and amyloidosis [4]. Adaptive immunity, as well as communication between central and peripheral immune systems, have also been implicated in the neuroinflammation associated with AD [5]. There is evidence to suggest that inflammation occurs before the clinical onset of AD, and several AD candidate genes for inflammation have been identified as well, further supporting this premise [6].

No treatment options are currently available to reduce or offset the risk posed by neuronal destruction in the progression of AD. The six drugs currently approved by the Food and Drug Administration (FDA), including Rivastigmine, Galantamine, Donepezil, Memantine, Memantine combined with Donepezil, and Tacrine, only offer symptomatic relief [1].

Keeping in view this central role of inflammation, various anti-inflammatory treatments in AD have shown efficacy with varying results after the conduction of many clinical trials. Non-steroidal anti-inflammatory drugs (NSAIDs) form the cornerstone of these treatments, which work mainly by inhibiting cyclo-oxygenase (COX) enzyme activity, which is responsible for the neuroinflammatory response prevalent in AD [3]. NSAIDs also maintain mitochondrial Ca2+ homeostasis and target Rho-GTPases (guanosine triphosphate) and peroxisome proliferator-activated receptors (PPARs); the latter pathways are associated with axon growth, tau protein phosphorylation, and astrocyte motility, all of which play a role in the pathogenesis of AD [7]. Furthermore, certain NSAIDs, including Sulindac, Ketorolac, Ibuprofen, and Naproxen among others, act as inhibitors of amyloid-β ( A-β) aggregation, a contributing event in the development of AD, by working as A-β fibril inhibitors in terms of predicted binding affinity [8]. While epidemiologic studies have shown a reduced incidence of AD with NSAIDs use, randomized clinical trials (RCTs) have failed to show promising results with NSAIDs in AD patients [3,9]. This, in part, has been attributed to the low number of RCTs conducted, the lack of a long-term, large-scale RCT using cognitive measures [3], and limitations in the studies held so far regarding duration and dose of NSAIDs used. Therefore, NSAIDs are currently not recommended for either prevention or treatment of AD [9].

This literature review aims to point out the discrepancies that exist between observational studies and RCTs regarding the role of NSAIDs in limiting the progression of AD. It also seeks to provide recommendations for the use of NSAIDs as well as novel anti-inflammatory drugs in AD patients. 

## Review

The earliest clinical trials to identify the role of NSAIDs in slowing down the progression of or preventing AD were conducted in the late 1990s. The most initial clinical indication about the possible role of anti-inflammatory drugs in AD came from a review of 17 epidemiologic studies, conducted to find out an association between arthritis, anti-inflammatory medications, and AD [10]. A clinical trial by Rogers et al., reviewing the impact of indomethacin in the progression of AD, revealed that 100-150 mg/day of indomethacin appeared to offer a protective role against cognitive decline in patients with mild-to-moderate AD when measured against a placebo group. The double-blind study, conducted over a duration of six months, assessed the efficacy of indomethacin by measuring percent change in cognitive behavior from the baseline, which was determined by several cognitive tests such as the Mini-Mental State Examination (MMSE), AD Assessment Scale (ADAS), Boston Naming Test (BNT), and Token Test (TK). Despite positive results, the study was limited by small sample size, as well as the short duration of time to fully recognize the spectrum of changes that can occur with AD [11]. However, a double-blind, placebo-controlled RCT of diclofenac/misoprostol in AD patients failed to reveal any beneficial effects in slowing progression or improving cognitive decline [12]. A larger, multicenter RCT, conducted over one year was also unable to show any positive impact on cognitive function by either naproxen or rofecoxib, indicating that patients with mild-to-moderate AD do not benefit from these NSAIDs compared to the placebo group [13].

The ADAPT (AD Anti-inflammatory Prevention Trial) study, conducted in a multicenter setting, was a placebo-controlled RCT to investigate the efficacy of NSAIDs in primary prevention of AD. Held over three years, after which the treatments were terminated following concerns over cardiovascular safety, the trial failed to show any benefit from celecoxib or naproxen in terms of prevention of AD over this duration. The study selected patients older than 70 years, with at least one first-degree relative with AD, and the outcome was determined as the diagnosis of AD in the selected patient, made by various tests for Dementia Evaluation (DE), laboratory testing as well as neuroimaging [14]. While this trial suggested that other NSAIDs, particularly the selective A-β-42 lowering agents (SALA) may be more beneficial in reducing the risk of AD, this was disproven by a pooled dataset from six prospective cohort studies, which showed that although NSAIDs use lowered the risk of AD, there was no significant difference between the SALA and the non-SALA groups [15]. An extended follow up of the patients included in the ADAPT study, conducted over almost two years, with subsequent collection and analysis of cerebrospinal fluid (CSF) from the participants, suggested that NSAIDs might even contribute to AD pathogenesis in its later stages. This seemed to occur more in patients with pre-existing AD pathology at the time of enrollment in the original ADAPT study, which, despite no clinical manifestations, influenced the impact of NSAIDs on AD pathogenesis. The study also hypothesized, in agreement with previously conducted observational studies, that NSAIDs may be protective against AD but only after two to three years of use. CSF biomarker results also supported the viewpoint that naproxen can be neuroprotective against cognitive deterioration in previously cognitively healthy individuals [16]. A follow-up trial, the Anti-inflammatory Prevention Trial followup study (ADAPT-FS), screened 1537 participants of the original ADAPT study by telephone assessment battery (TAB) as well as clinical examinations conducted at Dementia-evaluation visits (DEVs) seven years after the termination of the original research. The purpose of the trial was to examine the hypothesis suggested by the extended follow up of ADAPT study--whether the trend towards the decreased risk of AD with NSAIDs was sustained or not. The primary outcome in ADAPT-FS was the time to develop AD after enrollment in ADAPT. The trial diagnosed 89 cases of AD in addition to those diagnosed after ADAPT, taking the total toll to 161 AD patients. Combined data analysis from both studies failed to support the priorly reported decrease in AD incidence with naproxen over two to three years after use. An increased risk of death compared to the placebo group was also shown for celecoxib. The trial concluded that NSAIDs use could not be supported for AD prevention in the elderly, a result contrasting with evidence from epidemiological studies [17].

Certain theories have been propounded regarding the diverging relationship of NSAIDs with AD predicted by observational studies and RCTs. One holds that the results of observational studies may be spurious, arising due to some confounding or bias. Another suggests that the negative consequences in RCTs may be due to the notion that NSAIDs need to be taken for several years before the onset of AD to have a protective effect. As outlined above, the ADAPT and the ADAPT-FS trial do not support this claim; other RCTs, such as the Cache County study, reported a beneficial effect of NSAIDs only in certain groups, and after a certain period of use. Decreased incidence, as well as the prevalence of AD, occurred with NSAIDs use longer than two years; below this, no benefit occurred. The study also reported a link between the age of consumption and apolipoprotein E (APOE) E4 genotype: those who started at age less than 65 and were APOE E4 carriers derived more benefit from NSAID use [18]. The findings seemed to suggest that a window period exists after starting NSAIDs during which they do not impact the conversion risk to AD. While this is corroborated by the Rotterdam study [19], the ADAPT-FS trial does not agree with this presumption [20].

Some systematic reviews have also been carried out to compare the pre-existing data on NSAIDs use in AD. A meta-analysis of available RCTs carried out by Gupta et al. [21], compared seven placebo-controlled, double-blind trials, in which patients were evaluated using the MMSE or ADAS-cognitive (ADAS-Cog) scale. Both parameters failed to show any significant advantage of the use of NSAIDs over placebo for AD. The meta-analysis was limited by the short duration over which the RCTs were conducted (mostly <12 months), as well as defined sample size. It suggested that the relationship between NSAIDs and AD was inconclusive as of yet, and required further corroboration by larger, long-term RCTs [21].

Another systematic review and meta-analysis of 18 observational studies and an RCT, exploring the relationship between both NSAIDs (aspirin and non-aspirin) and steroids with AD, suggested that NSAIDs use provided an incident risk reduction of 28% for AD [3]. More extended NSAID use was linked with greater benefit, while no significant advantage was recorded for steroids. As with previous studies, there existed a discrepancy between the RCTs and the observational studies; however, this systematic review offered some explanations for the difference. According to this review, NSAIDs use is beneficial only for longer durations; the 15 months of NSAID use in the ADAPT trial due to risk-based interruption could have been insufficient to produce a sustained protective effect in the participants. Furthermore, the ADAPT trial faced a reduction in statistical power during its course, because the estimated incidence of 2.5% for AD upon which the hypothesis was based was greater than the actual prevalence of 1.1%. The review also suggested that since RCTs have not been conducted in specific populations with the APOE E4 genotype, results from RCTs cannot be considered the final word in determining the relationship between NSAIDs and AD [3]. The review recommends that NSAIDs, including aspirin if used over longer durations, can significantly lower the incident risk of AD. Since the review includes observational studies fulfilling at least five of eight Newcastle-Ottawa Scale (NOS) criteria and included newer studies, it was found to be statistically robust by sensitivity analyses.

A systematic review by Miguel-Alvarez et al., while not underscoring the role of NSAIDs in prevention of AD, suggested that NSAIDs have no role in the treatment of AD and should not be used as a therapeutic option. The review included seven carefully selected RCTs, based on MMSE, ADAS-Cog, and CDR-SOB (Clinical Dementia Rating Scale sum-of-boxes) parameters of evaluation. There was extensive heterogeneity among the studies included, not only in terms of NSAIDs administered, but also regarding dose, administration time, and follow-up duration. However, the lack of publication bias among the included studies strengthens the result of the review. The review concluded that there was no beneficial effect of NSAIDs on either cognition or overall disease severity [22]. Similar results were also reported by a Cochrane review of NSAIDs, aspirin, and steroids for treatment of AD, which included 14 RCTs. This review also reported a higher incidence of side effects, as well as a higher death rate among patients treated with NSAIDs, particularly COX-2 inhibitors [23].

While chronic NSAID use has been reportedly preferred over short-term use for prevention of AD [3], one review of both epidemiological and clinical trial studies hypothesizes that such usage might only be beneficial in the very early stages of AD pathogenesis, coincident with initial A-β deposition, microglial activation, and release of pro-inflammatory cytokines. Once the process of A-β deposition has started, NSAIDs are not useful; instead, a detrimental effect can occur owing to their inhibitory activity on already activated microglial cells. This hypothesis might in part explain why SALAs were more successful than non-SALAs, and why specific trials such as ADAPT failed to show an effect on AD prevention [24].

## Conclusions

A thorough review of the literature suggests conflicting opinions for the use of NSAIDs in AD. While observational and epidemiological studies have stressed on a beneficial role of NSAIDs in reducing the risk of AD or its progression, RCTs and meta-analyses thereof have failed to corroborate this significantly. No RCTs have been conducted to date in populations with APOE E4 genotype. For people with existing cognitive decline, as well as diagnosed AD, NSAIDs should not be administered, as no clinical evidence has been demonstrated regarding their benefit. The authors also recommend that further RCTs should be conducted over longer durations with larger samples to clarify the role of NSAIDs in the treatment of AD in selected populations.
